# Supplementary material for: Theoretical Approach for the Luminescent Properties of Ir(III) Complexes to Produce Red–Green–Blue LEC Devices
Source: Molecules. 2022 Apr 19;27(9):2623. doi: 10.3390/molecules27092623 (PMC9104581; doi:10.3390/molecules27092623)
Supplement: Supplementary file 1 [file molecules-27-02623-s001.zip › molecules-1664766-supplementary.pdf]

SUPPLEMENTARY MATERIALS

Theoretical approach about luminescent properties of Ir(III) complexes to produce red-green-blue LEC devices

Mireya Santander-Nelli,[1,2,*] Bastián Boza,[3] Felipe Salas,[3] David Zambrano,[4] Luis Rosales,[4] Paulina Dreyse[3,*]

[1]Advanced Integrated Technologies (AINTECH), Chorrillo Uno, Parcela 21, Lampa, Santiago, Chile.

[2]Centro Integrativo de Biología y Química Aplicada (CIBQA), Universidad Bernardo O'Higgins, General Gana 1702, Santiago 8370854, Chile.

[3]Departamento de Química, Universidad Técnica Federico Santa María. Avda. España 1680, Casilla 2390123, Valparaíso, Chile.

[4]Departamento de Física, Universidad Técnica Federico Santa María, Avda. España 1680, Casilla 2390123, Valparaíso, Chile.

[*]Corresponding authors: Mireya Santander-Nelli (ms@aintech.cl) and Paulina Dreyse (paulina.dreyse@usm.cl)

Table of content

S1. Benchmark study with other DFT functionals ……….…………..….. 3

S2. Geometries of the ground states and the triplet states………………... 5

S3. Energies of the molecular orbitals from HOMO-2 to LUMO+2, in the ground state.………………………..……………..………………...... 6

S4. Contribution to molecular orbitals from HOMO-2 to LUMO+2, in the ground state ……………………………………………..………....... 6

S5. Surface orbitals from HOMO and LUMO in the ground state ……… 7

S6. Molecular orbitals of the triplet excited state ………………………… 9

S7. Geometry parameters of ^{3}MC state …………………………………… 11

S8. Spin density of ^{3}MC state ...…………..……………………………….. 11

S9. Root-mean-square deviation (RMSD) between the ^{3}MLCT and ^{3}MC states………………………………………………………………………… 12

S10. Qualification scales of k_r, k_{nr}, *IP*, *EA* and $\Delta\lambda$ to choose RGB systems…. 12

S1. Benchmark study with other DFT functionals

In order to test the reliability of the selected methodology, a benchmark study was carried out for the **[Ir(ppy)$_2$(bpy)]$^+$** complex (ppy: 2-phenylpyridine, bpy: 2,2'-bipyridine). The absorption spectrum and emission energy were determined with five different DFT functional: B3LYP,[1,2] Cam-B3LYP,[3] BLYP,[2] M06-2x,[4] TPSSh[5] and PBE,[6] frequently used in Ir(III) complexes.

Table S1. Absorption and Emission wavelengths from ground and triplet excited states, respectively, for **[Ir(ppy)$_2$(bpy)]$^+$** (in dichloromethane).

	λ_{abs} (nm)	λ_{emi} (nm)
Experimental [7]	*250-310 (intense)*<br>*350-480 (broad)*	*598*
**B3LYP**	**250-300**<br>**350-410**	**643**
**Cam-B3LYP**	210-270<br>290-330	625
**BLYP**	300-390<br>430-530	942
**M06-2X**	200-270<br>300-340	506
**TPSSh**	260-340<br>390-430	730
**PBE**	290-360<br>430-520	929

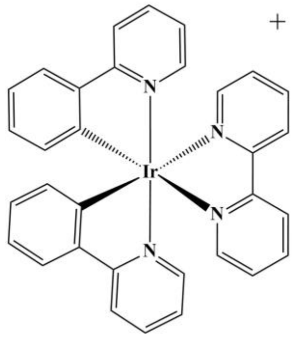

[Ir(ppy)$_2$(bpy)]$^+$

The absorption spectra were obtained from the optimized geometry at the ground state using the TD-DFT methodology and the T_1 state was fully optimized through TD-DFT optimization. The emission energy was calculated as the vertical energy difference between the relaxed triplet state and ground state at the optimized triplet geometry. The implicit solvent effects by the IEF-PCM method were also included using dichloromethane as solvent. The basis set and pseudo-potential used are the same described in the manuscript.

It is observed that the B3LYP and TPSSh functionals are the best methods to describe the absorption bands with a slight underestimation of the broad absorption bands at lower energies (350 to 480 nm) in comparison with the experimental data. Regarding the emission, all the tested methods showed an overestimation of the emission wavelength between 27 to 331 nm, however, the B3LYP and Cam-B3LYP functionals show to be more adequate in reproducing the λ_{emi} with small deviations of 45 and 27 nm, respectively, compared to the experimental data. In this way, the functional B3LYP is the only one able of properly describing the photophysical properties of both, the ground and the excited states of **$[Ir(ppy)_2(bpy)]^+$** complex, therefore, this pseudo functional was selected for the present study.

S2. Geometry parameters of the ground states and the triplet states

Table S2. Optimized geometric parameters of all complexes under study in the S_0 and T_1 states, determined at the B3LYP/6-31G(d)-LANL2DZ level of theory.

	1A		***1B***		***2B***		***2C***	
	S_0	T_1	S_0	T_1	S_0	T_2	S_0	T_2
Bond length (Å)								
Ir-C_1	2.016	2.015	2.021	1.993	2.019	2.006	2.023	2.021
Ir-C_2	2.031	1.983	2.024	2.006	2.022	2.020	2.019	2.008
Ir-N_1	2.077	2.076	2.074	2.072	2.073	2.041	2.088	2.096
Ir-N_2	2.202	2.225	2.307	2.245	2.292	2.311	2.285	2.291
Ir-N_3	2.325	2.243	2.309	2.274	2.295	2.296	2.287	2.307
Ir-N_4	2.083	2.089	2.082	2.090	2.081	2.092	2.074	2.042
Bond angle (deg)								
C_1-Ir-N_4	96.2	97.9	95.7	96.9	95.5	94.9	95.8	95.9
C_1-Ir-N_3	169.4	164.4	177.1	175.9	177.5	177.2	172.2	172.6
C_1-Ir-C_2	85.4	90.2	82.4	88.2	82.2	83.2	82.3	83.1
N_2-Ir-N_3	74.0	74.9	74.4	74.5	74.8	74.1	74.9	74.4
N_1-Ir-N_4	174.4	177.3	174.6	175.9	174.6	174.9	174.4	174.8
Diedral angle (deg)								
C_1- C_2-N_3-N_2	4.0	5.6	4.7	5.3	4.5	3.5	6.1	4.8
N_1- C_2-N_4-N_2	2.6	0.5	6.2	8.1	6.0	6.1	4.1	5.1

	3B		***3C***		***3D***		***3E***	
	S_0	T_2	S_0	T_3	S_0	T_2	S_0	T_2
Bond length (Å)								
Ir-C_1	2.015	2.002	2.013	2.018	2.013	2.000	2.012	2.019
Ir-C_2	2.012	2.021	2.017	2.023	2.013	2.000	2.016	2.025
Ir-N_1	2.085	2.065	2.078	2.077	2.084	2.082	2.075	2.073
Ir-N_2	2.287	2.199	2.279	2.232	2.194	2.179	2.293	2.223
Ir-N_3	2.284	2.244	2.277	2.234	2.194	2.179	2.281	2.215
Ir-N_4	2.077	2.096	2.092	2.094	2.084	2.083	2.089	2.098
Bond angle (deg)								
C_1-Ir-N_4	95.7	96.5	95.6	95.5	95.5	96.9	95.5	94.9
C_1-Ir-N_3	173.5	177.7	172.6	177.2	172.5	170.1	178.9	178.3
C_1-Ir-C_2	81.7	85.0	81.7	82.6	89.0	94.5	82.1	83.9
N_2-Ir-N_3	75.0	75.4	75.1	75.8	75.2	75.8	74.0	75.8
N_1-Ir-N_4	174.5	174.7	174.3	173.7	174.0	176.7	174.6	173.1
Dihedral angle (deg)								
C_1- C_2-N_3-N_2	4.9	5.2	6.3	5.0	4.2	4.7	4.7	6.3
N_1- C_2-N_4-N_2	3.6	6.7	3.4	3.9	7.6	7.9	7.1	7.5

S3. Energies of the molecular orbitals from HOMO-2 to LUMO+2, in the ground state

Table S3. Energy of frontier molecular orbitals (eV) and ΔHL of the ground state (S_0).

Complex	HOMO-2	HOMO-1	HOMO	LUMO	LUMO+1	LUMO+2	ΔHL
1A	-6.57	-6.42	-5.85	-2.85	-1.94	-1.85	3.00
1B	-6.49	-6.32	-5.85	-2.46	-1.94	-1.82	3.39
2B	-6.65	-6.58	-6.19	-2.54	-2.06	-1.95	3.65
2C	-6.67	-6.59	-6.20	-2.59	-2.33	-2.05	3.61
3B	-6.94	-6.92	-6.68	-2.66	-2.25	-2.15	4.02
3C	-6.99	-6.95	-6.71	-2.70	-2.43	-2.24	4.01
3D	-7.00	-6.86	-6.66	-2.65	-2.21	-2.12	4.01
3E	-6.97	-6.94	-6.73	-3.22	-2.28	-2.19	3.51

S4. Contribution to molecular orbitals from HOMO-2 to LUMO+2, in the ground state

Table S4. Contribution to molecular orbitals (%) of all complexes calculated from HOMO-2 to LUMO+2 in the ground state (S_0).

Complex	**HOMO-2**			**HOMO-1**			**HOMO**		
	**C^N**	**Ir**	**N^N**	**C^N**	**Ir**	**N^N**	**C^N**	**Ir**	**N^N**
1A	47.7	46.4	5.9	95.6	2.9	1.5	**58.4**	**39.5**	2.1
1B	42.9	51.1	5.9	96.2	1.5	2.4	**56.9**	**41.3**	1.7
2B	73.6	22.3	4.1	97.1	1.7	1.2	**59.1**	**39.0**	1.9
2C	76.7	19.3	4.0	97.6	1.5	1.0	**58.9**	**38.3**	2.8
3B	5.3	3.6	91.2	67.5	6.4	26.1	**60.7**	**33.4**	5.9
3C	37.4	25.0	37.7	96.6	2.6	0.8	**61.1**	**33.0**	5.9
3D	75.1	23.1	1.8	89.4	9.2	1.4	**61.7**	**36.5**	1.8
3E	31.0	22.8	46.2	64.4	8.8	26.9	**62.4**	**32.8**	4.9

Complex	LUMO			LUMO+1			LUMO+2		
	**C^N**	**Ir**	**N^N**	**C^N**	**Ir**	**N^N**	**C^N**	**Ir**	**N^N**
1A	0.7	2.2	**97.1**	94.7	4.0	1.3	89.7	4.3	6.0
1B	1.8	2.5	**95.8**	92.2	4.0	3.8	93.6	4.4	2.0
2B	2.1	2.5	**95.4**	91.8	4.1	4.1	93.5	4.6	1.9
2C	1.3	2.0	**96.7**	7.0	0.8	92.2	86.7	4.2	9.1
3B	2.6	2.7	**94.7**	92.0	3.9	4.1	94.1	4.6	1.3
3C	1.5	1.9	**96.6**	15.4	1.2	83.4	78.6	3.7	17.7
3D	0.8	2.1	**97.1**	94.6	4.2	1.2	94.8	4.0	1.2
3E	1.7	1.9	**96.4**	90.1	4.8	5.1	78.9	4.3	16.7

S5. Surface orbitals from HOMO and LUMO in the ground state

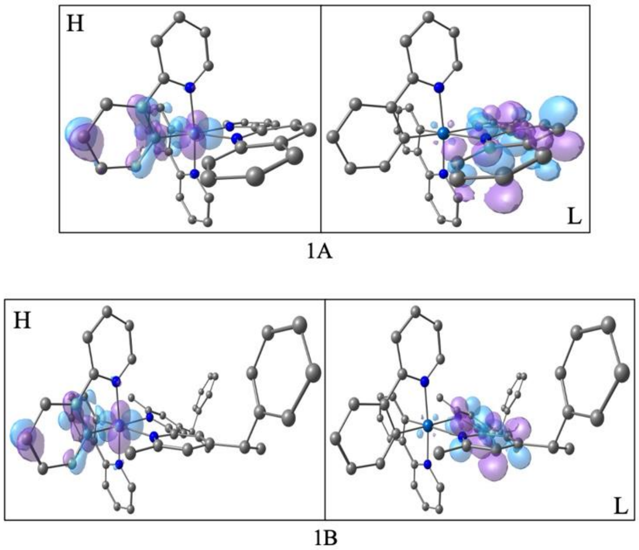

Figure S1. Surface of frontier molecular orbitals HOMO and LUMO (S_0) of series **1**.

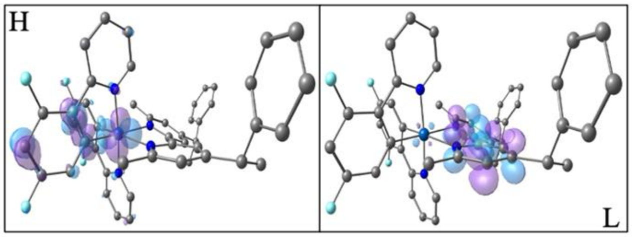

2B

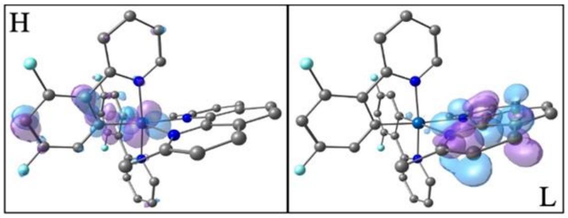

2C

Figure S2. Surface of frontier molecular orbitals HOMO and LUMO (S_0) of series **2**.

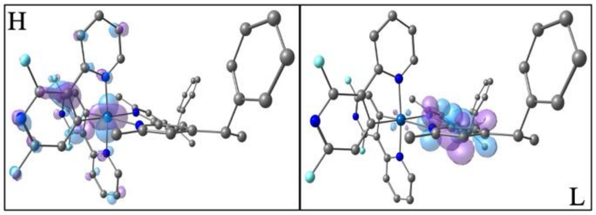

3B

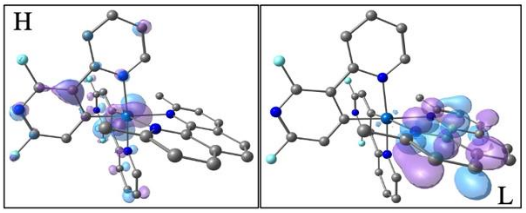

3C

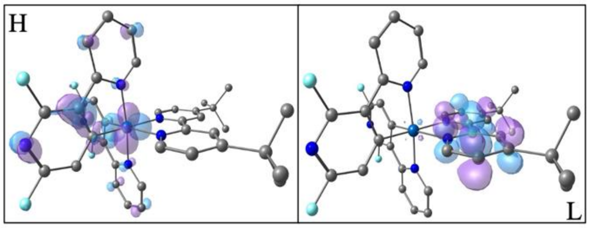

3D

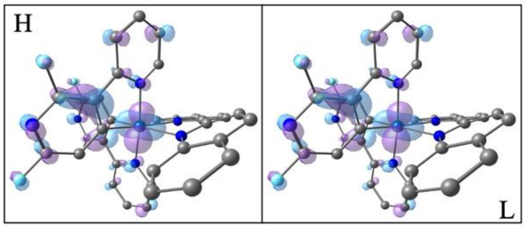

3E

Figure S3. Surface of frontier molecular orbitals HOMO and LUMO (S_0) of series **3**.

S6. Molecular orbitals of the triplet excited state

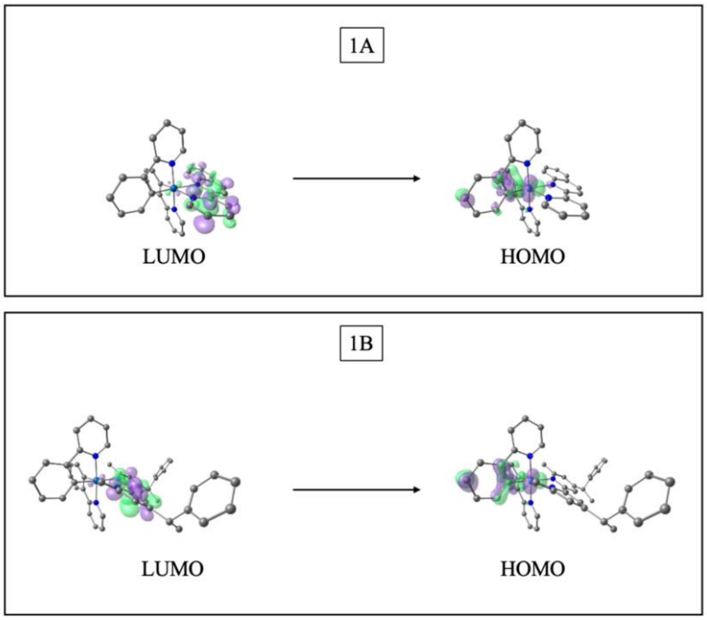

Figure S4. Frontier molecular orbitals involved in the radiative deactivation of the lowest-lying triplet excited state of series **1**.

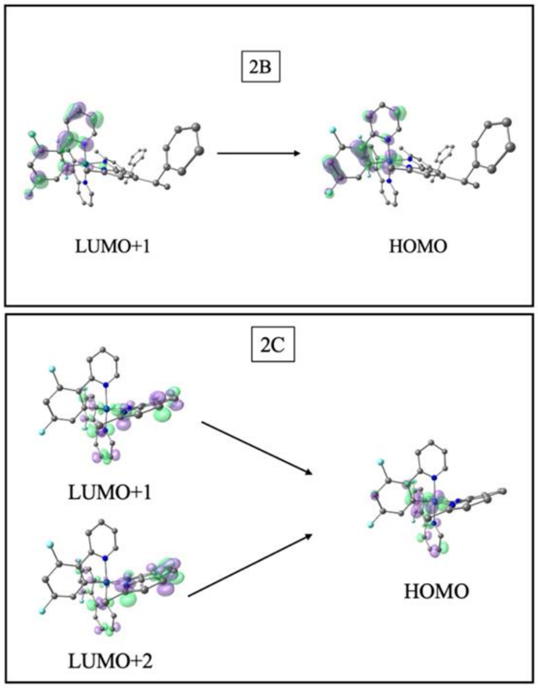

Figure S5. Frontier molecular orbitals involved in the radiative deactivation of the lowest-lying triplet excited state of series **2**.

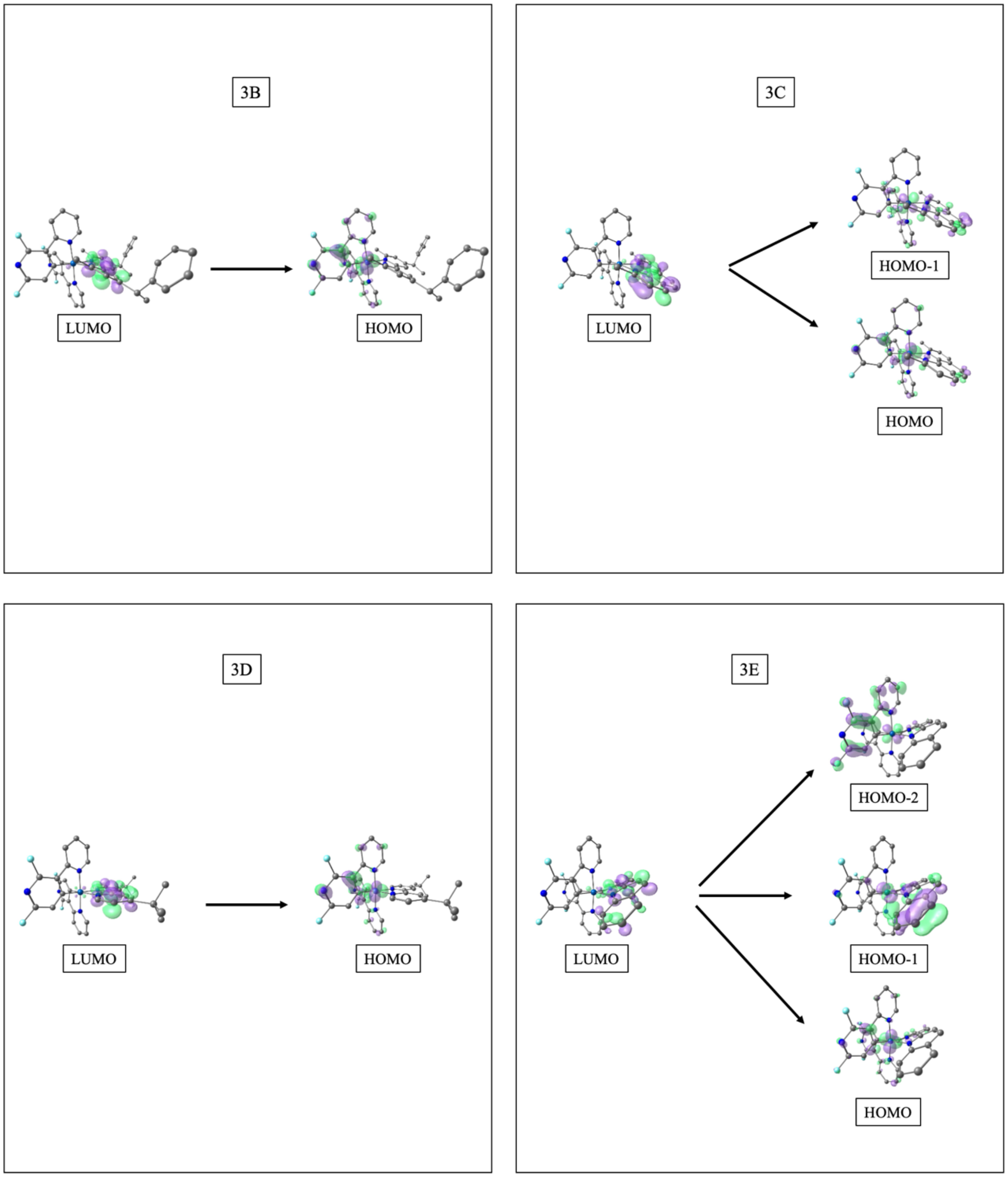

Figure S6. Frontier molecular orbitals involved in the radiative deactivation of the lowest-lying triplet excited state of series **3**.

S7. Geometry parameters of ^{3}MC state

Table S5. Selected geometric parameters of all complexes under study in the ^{3}MC state, determined at the B3LYP/6-31G(d)-LANL2DZ level of theory.

	1A	1B	2B	2C	3B	3C	3D	3E
Bond length (Å)								
Ir-C_1	2.02	2.03	2.03	2.03	2.02	2.02	2.03	2.02
Ir-C_2	1.99	2.05	2.05	2.05	2.04	2.01	2.03	2.05
Ir-N_1	2.08	2.23	2.21	2.22	2.22	2.10	2.29	2.20
Ir-N_2	2.22	2.36	2.36	2.37	2.35	2.27	2.25	2.39
Ir-N_3	2.23	2.26	2.26	2.26	2.25	2.26	2.20	2.25
Ir-N_4	2.09	2.69	2.63	2.62	2.64	2.08	2.58	2.59
Bond angle (deg)								
C_1-Ir-N_4	98.0	97.6	97.5	97.7	96.9	95.3	93.9	96.2
C_1-Ir-N_3	163.9	175.0	176.6	176.2	176.5	171.9	175.0	177.1
C_1-Ir-C_2	90.3	87.3	87.1	86.9	86.6	81.8	91.3	87.7
N_2-Ir-N_3	75.0	72.6	73.3	73.8	73.7	74.6	74.2	73.0
N_1-Ir-N_4	177.1	154.2	154.9	154.9	154.6	173.9	154.3	152.5
Dihedral angle (deg)								
C_1- C_2-N_3-N_2	5.8	33.3	30.3	29.5	30.5	5.9	23.0	29.7
N_1- C_2-N_4-N_2	0.2	11.8	10.7	9.8	10.8	4.0	12.9	13.0

S8. Spin density of ^{3}MC state

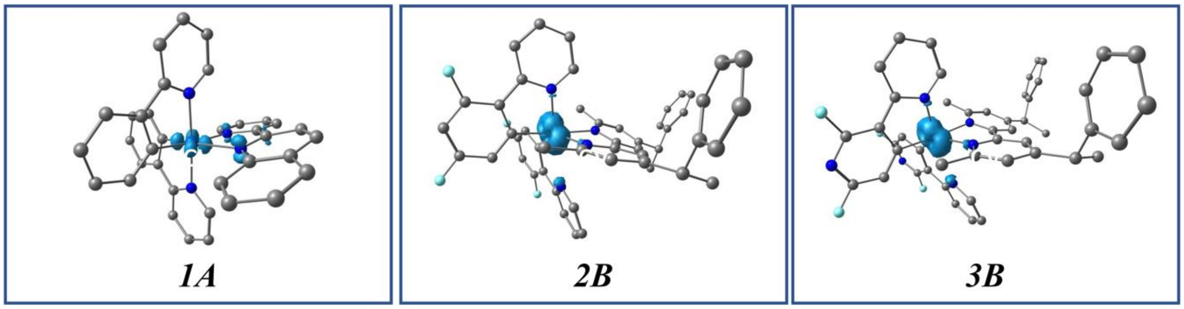

Figure S7. Spin density distribution in the optimized ^{3}MC state for representative complexes.

S9. Root-mean-square deviation (RMSD) between the ^{3}MLCT and ^{3}MC states

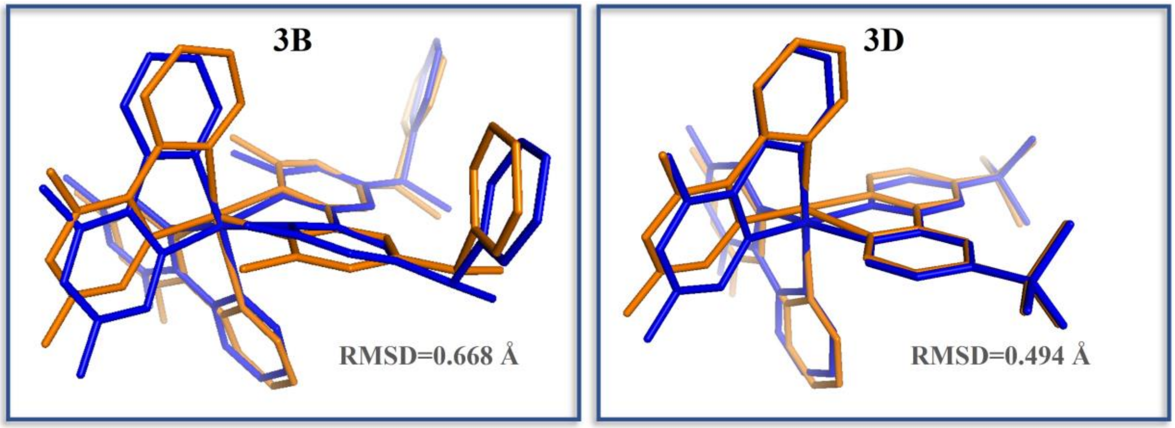

Figure S8. Superimposed structures of the 3MLCT states (bluen) and 3MC states (orange) for **3B** and **3D**, and their *RMSD* values.

S10. Qualification scales of k_r, k_{nr}, *IP*, *EA* and $\lambda\Delta$ to choose RGB systems

To build scales that would allow us to qualify the parameters studied as very favourable, favourable, or slightly favourable (or unfavourable in the case of k_{nr}), the calculated values of *IP*, *EA* and $\lambda\Delta$ were taken. In the case of k_r, an approximate calculation was made according to equation 2 described in section 3.4, considering the following expression: $k_r = (M^3\ \%LCT)\mu(^2{}_{S1})^2/\Delta E(S_1\text{-}T_n)^2$. Finally, in the case of k_{nr}, the values of $\Delta E(^3MC\text{-}^3MLCT)$ were considered as an approximation to identify whether or not this energy difference favours the radiative process of phosphorescence. The values calculated and determined according to this explanation are presented in Table S6. Table S7 shows the scales constructed to qualify each value as very favourable, favourable, or slightly favourable (or unfavourable in the case of k_{nr}).

Table S6. Photophysics and charge transport parameters to determine the best RGB systems.

**Complex**	**1A**	**1B**	**3E**	**2C**	**2B**	**3C**	**3B**	**3D**
**Color**	Red	Red	Green	Green	Green	Blue	Blue	Blue
λ_{em}/nm	701	599	574	548	547	454	448	440
k_r	211,600	538,756	3,844	25,600	47,089	5,041	685,584	229,441
k_{nr}	0.13	0.41	0.48	0.19	0.26	-0.26	-0.18	0.05
IP	5.85	5.85	6.75	6.21	6.20	6.72	6.70	6.67
EA	2.83	2.42	3.21	2.55	2.50	2.65	2.63	2.59
$\Delta\lambda$	0.13	0.27	0.20	0.18	0.30	0.21	0.32	0.27

Table S7. Building scales to quantify the photophysics and charge transport parameters to determine the best RGB systems.

**Parameter**	**Very favorable** +++	**Favorable** ++	**Slightly favorable** +	**Unfavorable** **0**
k_r	538,756 - 685,584	25,600 – 229,441	3,844 – 5,041	---
k_{nr}	≥ 0.48	0.26 – 0.48	0.05 – 0.19	-0.26 - -0.18
IP	5.85	6.20 – 6.21	6.67 – 6.75	---
EA	2.83 – 3.21	2.59 – 2.65	2.42 – 2.55	---
$\Delta\lambda$	0.13 – 0.21	0.27	0.30 – 0.32	---

References

(1) Becke, A. D. Density-Functional Thermochemistry. III. The Role of Exact Exchange. *J. Chem. Phys.* **1993**, *98* (7), 5648–5652. https://doi.org/10.1063/1.464913.

(2) Lee, C.; Yang, W.; Parr, R. G. Development of the Colle-Salvetti Correlation-Energy Formula into a Functional of the Electron Density. *Phys. Rev. B* **1988**, *37* (2), 785–789. https://doi.org/10.1103/PhysRevB.37.785.

(3) Yanai, T.; Tew, D. P.; Handy, N. C. A New Hybrid Exchange-Correlation Functional Using the Coulomb-Attenuating Method (CAM-B3LYP). *Chem. Phys. Lett.* **2004**, *393* (1–3), 51–57. https://doi.org/10.1016/j.cplett.2004.06.011.

(4) Zhao, Y.; Truhlar, D. G. The M06 Suite of Density Functionals for Main Group Thermochemistry , Thermochemical Kinetics , Noncovalent Interactions , Excited States , and Transition Elements : Two New Functionals and Systematic Testing of Four M06-Class Functionals and 12 Other Fun. **2008**, 215–241. https://doi.org/10.1007/s00214-007-0310-x.

(5) Shang, X.; Han, D.; Li, D.; Wu, Z. Theoretical Study of Injection, Transport, Absorption and Phosphorescence Properties of a Series of Heteroleptic Iridium(III) Complexes in OLEDs. *Chem. Phys. Lett.* **2013**, *565*, 12–17. https://doi.org/10.1016/j.cplett.2013.02.033.

(6) Adamo, C.; Barone, V. Toward Reliable Density Functional Methods without Adjustable Parameters: The PBE0 Model. *J. Chem. Phys.* **1999**, *110* (13), 6158–6170. https://doi.org/10.1063/1.478522.

(7) Suhr, K. J.; Bastatas, L. D.; Shen, Y.; Mitchell, L. A.; Frazier, G. A.; Taylor, D. W.; Slinker, J. D.; Holliday, B. J. Phenyl Substitution of Cationic Bis-Cyclometalated Iridium(III) Complexes for ITMC-LEECs. *Dalt. Trans.* **2016**, *45* (44), 17807–17823. https://doi.org/10.1039/c6dt03415b.
